# Inflammation-Related Immune-Modulatory SLURP1 Prevents the Proliferation of Human Colon Cancer Cells, and Its Delivery by *Salmonella* Demonstrates Cross-Species Efficacy against Murine Colon Cancer

**DOI:** 10.3390/pharmaceutics15102462

**Published:** 2023-10-13

**Authors:** Amal Senevirathne, Ram Prasad Aganja, Chamith Hewawaduge, John Hwa Lee

**Affiliations:** 1 College of Veterinary Medicine, Jeonbuk National University, Iksan 54596, Republic of Korea; amalsenevirathne@gmail.com (A.S.); rpaganja@gmail.com (R.P.A.);; 2 Institute of Animal Transplantation, Jeonbuk National University, Iksan Campus, Iksan 54596, Republic of Korea

**Keywords:** α7-nAChR, SLURP1, colorectal cancer, receptor inhibitor, cell cycle, *Salmonella*

## Abstract

This study investigates the anticancer properties of the α7-nAChR antagonist SLURP1 with a specific focus on its effect as an inflammation modulator on human colorectal cancer cell lines Caco2, Colo320DM, and H508 cells. The investigation includes the evaluation of cell cycle arrest, cell migration arrest, endogenous expression of SLURP1 and related proteins, calcium influx, and inflammatory responses. The results demonstrate that SLURP1 not only inhibits cell proliferation but also has the potential to arrest the cell cycle at the G1/S interface. The impact of SLURP1 on cell cycle regulation varied among cell lines, with H508 cells displaying the strongest response to exogenous SLURP1. Additionally, SLURP1 affects the nuclear factor kappa B expression and effectively reverses inflammatory responses elicited by purified lipopolysaccharides in H508 and Caco2 cells. This study further confirmed the expression of human SLURP1 by *Salmonella,* under *Ptrc* promoter, through Western blot analysis. Moreover, *Salmonella* secreting SLURP1 revealed a significant tumor regression in a mouse CT26 tumor model, suggesting the cross-species anticancer potential of human SLURP1. However, further investigations are required to fully understand the mechanisms underlying SLURP1’s ability to prevent cancer proliferation and its protective function in humans.

## 1. Introduction

The relationship between inflammation and colon cancer is well established. Research spanning pharmacological, genetic, and epidemiological studies has demonstrated a strong link between persistent inflammation and tumor development [1]. Anti-inflammatory agents such as 5-aminosalicylic acid are the first line of treatment for colon inflammation/ulcerative colitis [2] that targets peroxisome proliferator-activator receptor-γ. The manipulation of anti-inflammatory signaling via alpha 7 nicotinic acetylcholine receptor (α7-nAChR) is a plausible approach to reduce the synthesis of NF-kB transcriptional factor and inflammatory cytokines such as TNF- α and IL-1β. In this prospect, nicotine can be used as a treatment; however, its high toxicity on cells is a considerable limitation. Recent studies have identified secreted mammalian Ly-6/urokinase-type plasminogen activator receptor-related protein 1 (SLURP1) as a potent α7-nAChR antagonist that can significantly suppress epithelial cancer proliferation even at a concentration below 1 nM [3]. Therefore, exploring SLURP1’s impact on colon cancer proliferation and anti-inflammatory effects could broaden its therapeutic applications.

SLURP1 can efficiently scavenge on plasminogen activator urokinase (PLAU) and block PLAU receptors [4]. Activation of plasminogen could promote cancer cell proliferation [5,6]; thus, inhibition of this pathway by SLURP1 could have huge implications on cancer inhibition. The SLURP1 is structurally homologous to snake and frog neurotoxins [7,8,9] and competitively binds with α7-nAChRs. A rare point mutation of the SLURP1 gene leads to a disease condition called Mal De Meleda, which causes dramatic morphological changes in skin epithelial cells [10,11]. Most tissues express SLURP1 constitutively [12,13,14] without distinguishing between cancerous and non-cancerous cells. Analysis from clinical cohort studies reflects that high SLURP1 patients had longer survival periods compared to SLURP1 low patients [15]. Thus, it is hypothesized that SLURP1 plays an essential role in tissue/cellular homeostasis by modulating multiple regulatory pathways [7,8], particularly its direct interaction with α7-nAChRs, which are membrane-bound ion channels controlling Na^+^ and Ca^2+^ efflux in cells. This interaction may trigger downstream signaling that affects cellular proliferation [16]. With these perspectives, SLURP1 could have therapeutic implications for managing inflammation in colorectal cancer. Thus, as an autocrine/paracrine signaling molecule, SLURP1 could hold future therapeutic applications in colorectal cancer.

In addition to its anti-inflammatory role, SLURP1 is of prime interest for its anti-tumor properties supported by its ability to inhibit cell proliferation [3]. To evaluate its anticancer effect in an animal model, we employed a CT26 tumor model in BALB/c mice. To target tumor tissues effectively while minimizing side effects, we employed a live attenuated *Salmonella* delivery system transformed with a plasmid for constitutive expression of recombinant SLURP1 facilitated by a beta-lactamase secretory signal. The attenuation of the *Salmonella* delivery strain demands deletion of virulent genes such as the *lon* gene (encodes Lon protease) that regulates the expression of early virulence genes required for bacterial pathogenesis [17] and the *cpxR* gene (encodes CpxR protein) that promotes virulence by efficient adhesion [18].

In this study, we sought to investigate the anti-tumor effect inflicted by *Salmonella* Typhimurium (ST) secreting SLURP1 in BALB/c mice implanted with CT26 tumors. The designed therapeutic strain was administered to elucidate the cross-species anti-tumor potential of recombinant human SLURP1 in tumor-bearing mice. As a result, using such a target-oriented delivery of anti-inflammatory peptide, SLURP1 shows promise for future cancer treatments. It may be necessary to conduct further studies to gain a more comprehensive understanding of SLURP1 in human tumor models.

## 2. Materials and Methods

### 2.1. Cells and Reagents

The Caco2 cell line was acquired from American Type Culture Collection (ATCC: CCL-2; Manassas, VA, USA) and cultured in Dulbecco’s Modified Eagles Medium (DMEM; Lonza, Walkersville, MD, USA), 20% fetal bovine serum (FBS; Serana, Gmbh, Pessin, Germany) and 1% broad-spectrum antibiotics (Thermo Scientific, Waltham, MS, USA). Cells were grown in a humidified 5% CO_2_ atmosphere at 37 °C. The Colo320DM and H508 cells were generously provided by Dr. Eun-Jung Jin at Wonkwang University, South Korea, and maintained in Roswell Park Memorial Institute Medium 1640 (RPMI 1640; Thermo Scientific, Waltham, MS, USA), 10% FBS, and 1% broad-spectrum antibiotic. Mouse colon cancer cell line CT26 was purchased from American Type Culture Collections (ATCC; CRL-2638: RRID: CVCL_7256) and propagated on DMEM medium.

### 2.2. Construction of Rough ST Mutant Secreting Human SLURP1 Protein

The mRNA coding sequence of the human *SLURP1* gene, accession number AY579079.1, (312 bp) retrieved from the National Center for Bioinformatics (NCBI) database was codon-optimized for ST and chemically synthesized (Bioneer, Daejeon, Republic of Korea). The synthesized gene was cloned in the *asd* complemented pJHL65 plasmid under the direct control of the *Ptrc* constitutive expression promoter. Subsequently, the cloned plasmid (pJHL65::SLURP1) was electro-transformed into ST strain JOL1800 (Δ*lon* Δ*cpxR* Δ*rfaL* Δ*asd*), designated as JOL2238. The expression of the recombinant protein by JOL2238 was confirmed by an immunoblotting assay [19] using an HRP-labeled anti-his tag antibody (Bio-Rad; Cat: MCA5995P Oxford, UK). For protein purification purposes, the recombinant gene was cloned and expressed in the pET28a system (Novagen, Madison, WI, USA) by *E. coli* (DE3) transformation. The protein was purified according to the native purification procedure as described previously [20]. The purified protein fraction was buffer exchanged to phosphate-buffered saline (PBS) using buffer exchange columns (GE Healthcare, Buckinghamshire, UK) and concentrated using protein concentrator columns (Amicon Ultra 4, Millipore, Tullagreen, Ireland) with a 10 kDa cutoff limit. Protein concentrations were measured using the Bradford assay [21] and stored at −80 °C until use. The amino acid sequences of human SLURP1 and its related proteins, including human SLURP2, Human Lynx1, Human Lypd6, Mouse SLURP1, Neurotoxin 1, Erabutoxin, and α-Bungarotoxin were retrieved from the NCBI database and analyzed using Mega 7.0 software to assess the degree of sequence conservation [22].

### 2.3. SLURP1-Derived Cancer Cell Proliferation Inhibition

Colon cancer cell lines Caco2, Colo320DM, and H508 cells were propagated in 96-well plates in replicates (Axygen; Merck, Darmstadt, Germany) at 1 × 10^4^ CFU/well. Cells were treated with recombinant SLURP1 (rSLURP1) protein at 0, 10, 50, 100, and 200 nmol/well concentration and incubated at 37 °C, 5% CO_2_ for 72 h, The cell proliferative response was assessed using MTT [3-(4,5-dimethylthiazol-2-yl)-2,5-diphenyltetrazolium bromide] following a previously established procedure [23] and compared against the non-treated control. Growth-related experiments were conducted in triplicate. Periodical images were captured using the bright field microscope.

### 2.4. Scratch Wound Healing

The rSLURP1’s effect on cell proliferation and migration was tested by in vitro scratch wound healing assay [24]. Briefly, colorectal cancer cell lines (Caco2, Colo320DM, and H508 cells) were cultured to 60% confluent monolayer in 24-well plates, and scratches in the cell were created using a sterile 200 µL volume pipette tip. Then, cells were exposed to 200 nM/well rSLURP1 or medium alone. Cells were periodically observed using a Phase-contrast microscope (Leica, Leitz Park, Wetzlar, Germany), and images were documented after 3 h and 17 h of initial treatment. Gap closure was measured to evaluate the anti-proliferative effects.

### 2.5. SLURP1-Induced Morphological Changes in Cancer Cells

SLURP1-induced morphological changes were evaluated using the IncuCyte live imaging system (Essen BioScience; Ann Arbor, MI, USA) in Caco2 cells cultured in 96-well plates at 1 × 10^4^ cells/well. Cells were treated with rSLURP1 at 50, 200 nM and examined to investigate SLURP1-induced changes in cell morphology and cell proliferation for three days.

### 2.6. Effect of rSLURP1 on Endogenous Expression

The cellular effect of exogenous rSLURP1 on endogenous SLURP1 and α7-nAChR was evaluated on H508 cells (highest inhibited) and Caco2 (least inhibited) cells. Cells cultured in 24-well plates were treated with rSLURP1 at 200 nM/well for 24 h. Total RNA was extracted, and cDNA was prepared. Quantitative real-time (qRT-PCR) analysis was performed to determine the relative expression level of SLURP1 and α7-nAChR following the standard procedure. In addition, the level of secreted SLURP1 concentrations was evaluated by indirect enzyme-linked immunosorbent assay (ELISA). Briefly, cells were treated with 100 nM/well for 24 h, and the supernatants were collected and filtered. The filtered supernatants were concentrated 10 times using Amicon concentrator columns and directly coated into ELISA plates in 0.5 M Na_2_CO_3_/NaHCO_3_ coating buffer. The captured SLURP1 molecules were detected using SLURP1-specific rabbit polyclonal antibody (1:3000) (prepared in the lab by inoculating SLURP1 in rabbits). For color development, HRP-conjugated anti-rabbit antibody was used (1:5000).

### 2.7. Calcium Influx Assay

Caco2 cells grown at 80% confluent monolayer were washed twice in Krebs-Ringer buffer with or without 2.6 mM CaCl_2_ [25] and incubated for 1 h at 37 °C. Then, monolayers were washed with a pre-warmed growth media and further incubated for 1 h for recovery. To investigate Ca^2+^ influx, pre-treated cells were incubated with Fluo-3AM calcium indicator (Invitrogen, Waltham, MA, USA) per the manufacturer’s instructions and dissolved in Krebs-Ringer buffer. The cells were washed twice with the same buffer and quantified by flow cytometry (Miltenyi Biotec, Bergisch Gladbach, Germany) after 30 min in the dark.

### 2.8. qRT-PCR Assay

Colorectal cancer cells (Caco2, Colo320DM, and H508 cells) from SLURP1 exposure experiments were used to isolate total RNA by a commercial RNA extraction kit (GeneALL; Dongnam-ro, Seoul, Republic of Korea) following the manufacturer’s instructions. RNA (1 μg) was converted to cDNA (TOYOBO, Tokyo, Japan). The relative gene expression was quantified in ABI (Step One Plus, Applied Biosystems, Waltham, MA, USA) using a SYBR green PCR master mix (Elpis Biotech, Daejeon, Republic of Korea) with primers listed in Table S1. Gene expression data were normalized to glyceraldehydes-3-phosphate dehydrogenase (GAPDH) and quantified as fold change 2^−ΔΔCT^ relative to the control [26]. The data obtained from three trials were analyzed and expressed as mean ± SD.

### 2.9. Effect of SLURP1 under Inflammation

The influence of SLURP1 on the expression of pro-inflammatory cytokines was determined by qRT-PCR. Caco2, Colo320DM, and H508 cells cultured at 70% confluent monolayer were treated with 200 ng/well purified *Salmonella* lipopolysaccharides with or without SLURP1 at 200 nM/well. After 24 h incubation, cells were harvested for total RNA isolation and cDNA preparation. The expression level of TNF-α, IFN-γ, and IL-1β was analyzed. The reversal of LPS-induced cell proliferation was assessed by MTT assay, 24 h post incubation, as described before using cells exposed to medium, SLURP1 alone, LPS alone, or SLURP1 and LPS in combination.

### 2.10. SLURP1-Induced Cell Cycle Analysis

The effect of recombinant SLURP1 on cell cycle arrest was investigated by flow cytometry analysis. Caco2, Colo320DM, and H508 cells cultured in 96-well plates were treated with rSLURP1 protein at 200, 1000, and 2000 nM/well for 24 h in a humidified, 5% CO_2_ atmosphere. After treatment, cells were stained with propidium iodide and prepared for flow cytometer analysis. Briefly, cells were fixed with 70% ethanol for 30 min at 4 °C followed by PBS wash. Cells were treated with RNases (Qiagen, Hilden, Germany), and the cell populations in the G1, S, and G2/M phases were quantified. The experiment was repeated twice, and the average values were considered for the analysis.

### 2.11. SLURP1 Effect on Cell Cyclins

The effect of SLURP1 on the cell cycle of Caco2, Colo320DM, and H508 cells was assessed by expression analysis of selected cell cyclins, Cyclin E, Cyclin D1, Cyclin D2, Cyclin D3, Cyclin B1, Cyclin B2, and Cyclin A2. Cells grown at 60% confluent monolayer were treated with 200 nM/well rSLURP1 for 24 h. The total RNA was extracted from cells and converted to cDNA. As described above, the relative fold change expression of cell cyclins was quantified by qRT-PCR.

### 2.12. Western Blot

The secretion of recombinant SLURP1 by ST JOL2238 carrying a therapeutic plasmid (pJHL65::SLURP1) was evaluated using the Western blotting technique. The bacterial culture grown overnight at 37 °C was sub-cultured for 3–4 h, harvested, and sonicated 2–4 cycles for 5 s with a 60 s gap at 50% amplitude. The supernatant was collected, and SLURP1 was detected by Western blot using an HRP-labeled anti-his tag antibody (1:4000). The antibody-treated Polyvinylidene fluoride membrane was incubated with chemiluminescence reagent (Westsave Gold, AbFrontier, Seoul, Republic of Korea) to develop a film. The developed images were documented for analysis (Cytiva, Marlborough, MA, USA).

### 2.13. Anti-Tumor Effect in Mouse Model

Five-week-old female BALB/c mice (*n* = 8) were purchased from Samtako Korea (Seorang-ro, Osan, Republic of Korea). Mice were induced for tumors by implanting 10^6^ cells/50 μL CT26 cells in DMEM on the lower back through a subcutaneous route. After two weeks, 100–150 mm^3^ tumors were obtained and subjected to PBS and rough ST JOL1800 with the empty plasmid or the therapeutic plasmid (1 × 10^7^ CFU/mouse) inoculation via the intraperitoneal route. A booster inoculation was applied 9 days after the primary inoculation. The change in tumor volume was measured for three weeks. At the end of the experiment, tumors were excised and proceeded for RNA extraction. The expression level of cell cycle and tumor-related genes were quantified by qRT-PCR. All animal studies were carried out under standard animal welfare conditions and were approved by the Jeonbuk National University Animal Ethics Committee (accession number: JBNU-2021-027), following the guidelines of the Korean Council on Animal Care and Korean Animal Protection Law, 2007; Article 13.

### 2.14. Statistical Analysis

All experiments were carried out at least three times. The results were expressed as mean ± SD. Statistical significance was determined using Student’s *t*-test. Differences were considered significant if the calculated *p*-value was <0.05. Statistical analysis was conducted in GraphPad Prism 9.0 Software, La Jolla, CA, USA. Each experiment was conducted in triplicates.

## 3. Results

### 3.1. SLURP1 Inhibits Cell Proliferation

**Figure 1:**
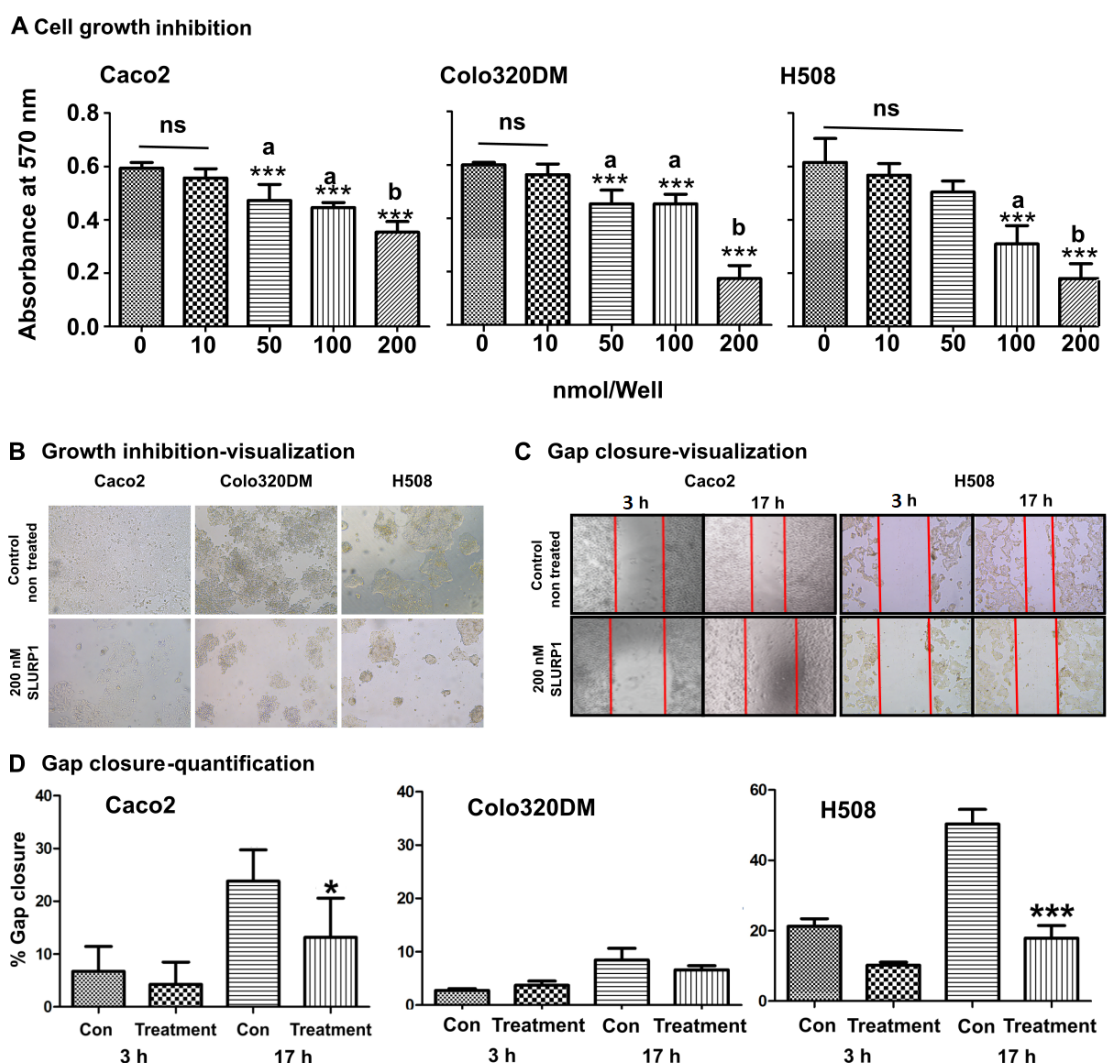
SLURP1 mediated cell proliferation and migration arrest. (**A**) The viability of colon cancer cell lines was assessed by rSLURP1 treatment at variable concentrations for 72 h. Data were analyzed by Student’s *t*-test, and statistically significant differences against the 0 nM/well control were indicated as *** at *p* < 0.001, ns = non-significant difference. Data were presented as mean ± SD, *n* = 5. Different letters (a and b) denote significant differences, while the same letter indicates no significant difference among the groups. (**B**) Microscopic images showing after 7 days of SLURP1 exposure at 200 nM/well concentration. (**C**) Gap closure of colon cancer cell lines after 200 nM/well exposure at 3 h and 17 h time points. (**D**) Quantification of gap closure. The change in % gap closure was calculated against the corresponding gap before treatment. A statistically significant difference is indicated by * at *p* < 0.05 and by *** at *p* < 0.001, *n* = 5, and data represent mean ± SD.

The effect of rSLURP1 on cell proliferation was investigated using purified rSLURP1 protein with Caco2, Colo320DM, and H508 cells at different concentrations (Figure 1A). SLURP1 treatment resulted in a dose-dependent cell proliferation inhibition, with a significantly low cell count observed in cells treated with 100 nM/well and 200 nM/well of SLURP1. Approximately 55% inhibition (about 33% for Caco2 and 66% for both Colo320DM and H508 cells) was observed at 200 nM/well compared to the non-treated control (Figure 1A,B); this was evident in visual observation under the phase-contrast microscope indicating that there was no increase in characteristic apoptotic phenotype in cells. However, a significant reduction in cell number was evident. Further, SLURP1-induced cellular effect on cell proliferation and migration was examined by scratch wound healing assay on cells grown to confluent monolayer (Figure 1C,D). Straight scratches made on complete confluent monolayers were exposed to 200 nM of rSLURP1 and observed under the phase-contrast microscope. Time-dependent percentage gap closure was monitored and revealed that there was a significant inhibition in gap closure in all wells treated with rSLURP1, implying the potential cell migration inhibition by SLURP1 (Figure 1D). The effect was more pronounced in H508 cells.

### 3.2. SLURP1’s Effect on Cancer Cell Morphology and Proliferation

**Figure 2:**
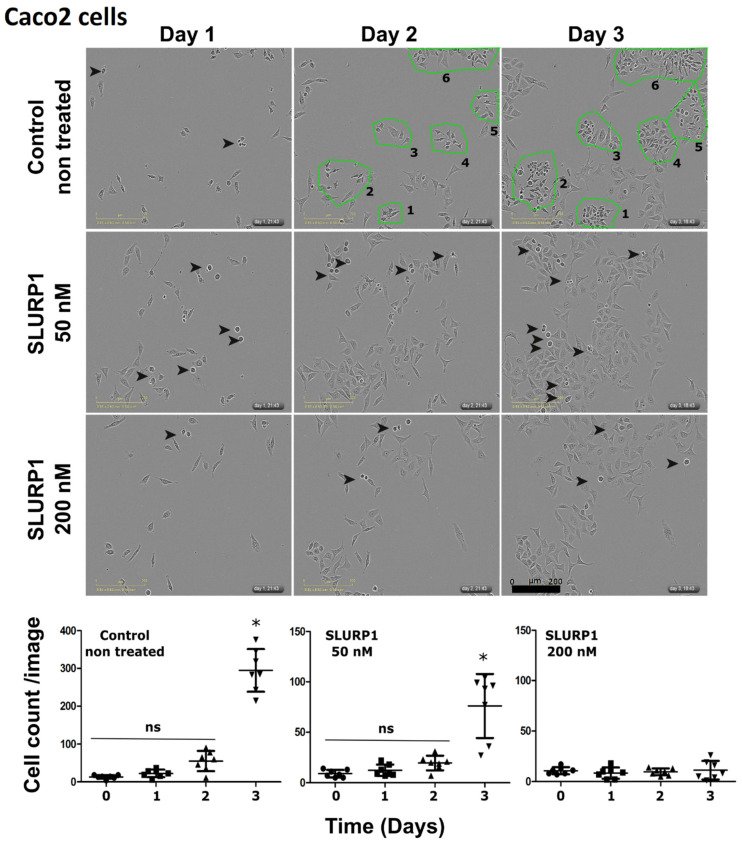
Morphological changes upon SLURP1 exposure. Caco2 cells were exposed to rSLURP1 treatment at 50 and 200 nM concentrations. Arrows indicate cells with rapid cell proliferation phenotypes. The green color enclosure indicates the rapidly dividing cells. The number of cells was quantified per image field and presented for numerical demonstration of cell proliferation. Data were analyzed by Student’s *t*-test. The error bars denote the mean ± SD, ns = non-significant, * *p* < 0.05. Images obtained were presented in a scale bar of 200 μM.

SLURP1-treated Caco2 cells were investigated under the IncuCyte real-time imaging system. During the 3-day observation period, differentiation of rapidly proliferating cells could be observed in non-treated cells (Figure 2). However, the rate of cell proliferation was significantly low in SLURP1-treated cells in a dose-dependent manner, higher at 200 nM concentrations. Under test conditions, no significant cell death was evident, confirming that the SLURP1 is not cytotoxic on cell culture.

### 3.3. Effect of Recombinant SLURP1 on Endogenous Expression

**Figure 3:**
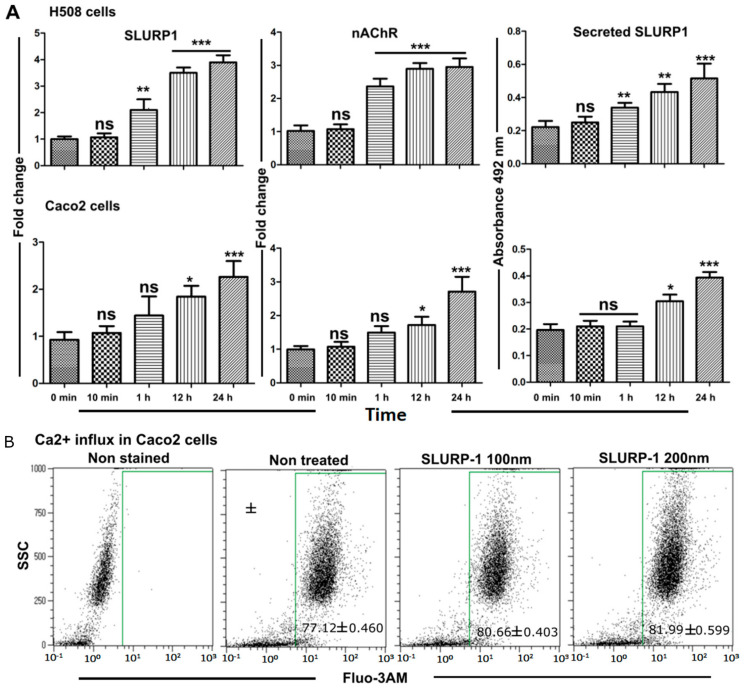
Endogenous expression of SLURP1, α7-nAChR, secretion of SLURP1, and calcium influx. (**A**) Endogenous SLURP1 and α7-nAChR expression and SLURP1 secretion were measured upon SLURP1 treatment. Data were analyzed by Student’s *t*-test, and statistically significant differences against the 0 min control were indicated as * *p* < 0.05, ** *p* < 0.01, *** *p* < 0.001, and ns = non-significant difference. Data were presented as mean ± SD, *n* = 5. (**B**) Calcium influx was quantified by Fluo-3AM mediated flow cytometry analysis, *n* = 5, and mean ± SD was indicated in each dot plot.

The inhibitory effect of SLURP1 can occur due to changes in expression levels of cholinergic receptors such as nAChRs; this may also be influenced by endogenous SLURP1 levels. To fully understand the expression of these genes, nAChR and SLURP1 were assessed at the mRNA level using qRT-PCR. For this, the effect of SLURP1 treatment on H508 and Caco2 cells was investigated at the mRNA level. A significant difference in endogenous expression of SLURP1 and α7-nAChR was evident in H508 cells than in the Caco2 cells. Furthermore, the quantity of secreted SLURP1 in the cell culture supernatant was examined, revealing an increased secretion of SLURP1 into the culture supernatants in both types of cells stimulated by an exogenous source. Such secretion may act as autocrine/paracrine signaling molecules to affect adjacent cells (Figure 3A). It was also observed that Caco-2 cells treated with rSLURP1 increased Ca^2+^ influx when reacted with the Fluo-3AM calcium indicator. Approximately 4–6% increase in Fluo-3AM positive cell response was observed in a dose-dependent manner. Such enrichment in an influx of Ca^2+^ ions by SLURP1 treatment probably indicates a response to the over-expression of α7-nAChR ion channels (Figure 3B).

### 3.4. SLURP1-Induced Anti-Inflammatory Responses

**Figure 4:**
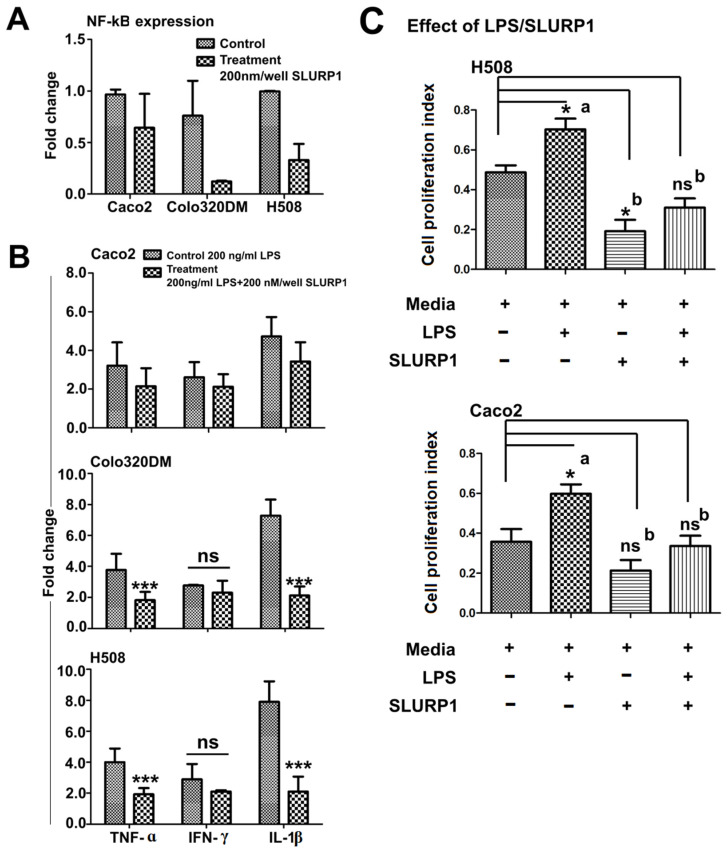
NF-kB expression, inflammatory cytokine expression, and abolishment of LPS effect by SLURP1. (**A**) The expression of NF-kB level as estimated by qRT-PCR, (**B**) SLURP1 mediated reversal of LPS induced inflammation measured through TNF-a, IFN-γ, and IL-1β, (**C**) SLURP1 mediated reversal of LPS induced cell proliferation. Data were analyzed by Student’s *t*-test, and statistically significant difference against the non-treated control was represented as ns = non-significant difference, * *p* < 0.05, *** *p* < 0.001, *n* = 5, and mean ± SD. Different letters (a and b) denote significant differences, while the same letter indicates no significant difference among the groups.

The treatment of the SLURP1 protein with Caco2, Colo320DM, and H508 cells revealed significant modulation in the expression of NF-kB [27]; this leads to reduced inflammatory cytokine production as marked by significantly lower expression of TNF-α, IFN-γ, and IL-1β in the treated cells (Figure 4A,B). H508 and Colo320DM responded substantially, while Caco2 had minimal inflammatory cytokine reduction. Also, the amount of SLURP1 secreted by cells increased over time after its exogenous supply (Figure 3A); this could be correlated with the reduction in inflammatory cytokines observed 24 h after SLURP1 treatment. Furthermore, SLURP1 was able to abort LPS-mediated cell proliferative responses in both H508 and Caco2 cells (Figure 4C).

### 3.5. SLURP1-Induced Cell Cycle Arrest

**Figure 5:**
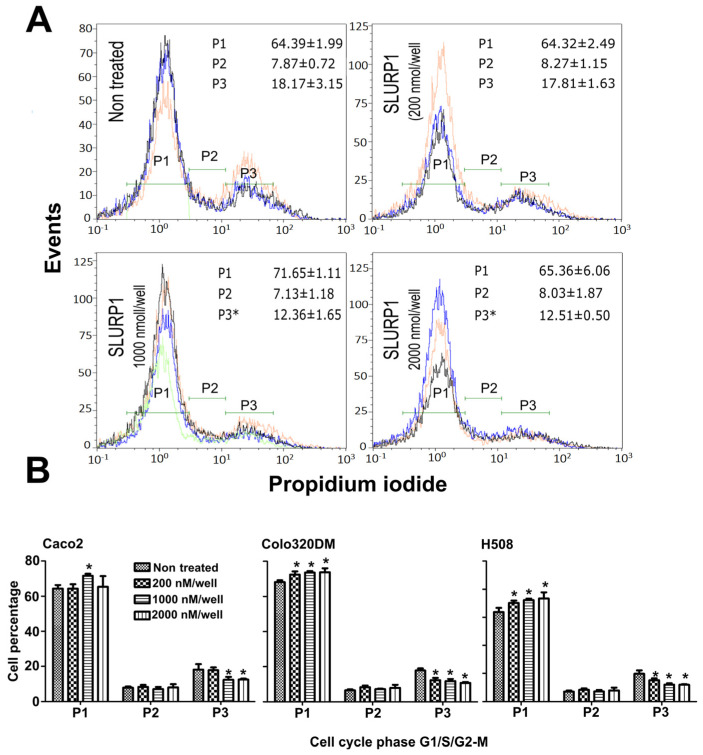
Assessment of cell cycle arrest by SLURP1 treatment. (**A**) Representative histogram of flow cytometric analysis of cell cycle phases of Caco2 cells treated with SLURP1 by propidium iodide staining method. Each P1, P2, and P3 phase indicates the G1, S, and G2 phases of the cell cycle. (**B**) Graphical representation of quantification of each cell cycle phase under SLURP1 treatment with different concentrations. Data were analyzed by Student’s *t*-test, and the significant difference against the non-treated control was determined at * *p* < 0.05, *n* = 5. The error bar indicates mean ± SD.

The potential impact of SLURP1 on the cell cycle phase was investigated using flow cytometry following exposure to concentrations ranging from 200 nM to 2 μM. The purified SLURP1 treatment induced a dose-dependent inhibition in cell proliferation and did not induce apoptosis in 24 h observation period. Interestingly, recombinant SLURP1 treatment elucidated cell cycle arrest at the G1 phase (Figure 5A). At all the tested concentrations of SLURP1, there was prominent cell cycle arrest at the P1 transition phase (G0/G1 transition phase) for Colo320DM and H508 cells, while such arrest was noted at higher concentrations for Caco2 cells. There was a substantial reduction in cells at the G2/M phase with an increase in SLURP1 protein concentration for all the tested cell lines (Figure 5B). 

### 3.6. qRT-PCR Analysis

**Figure 6:**
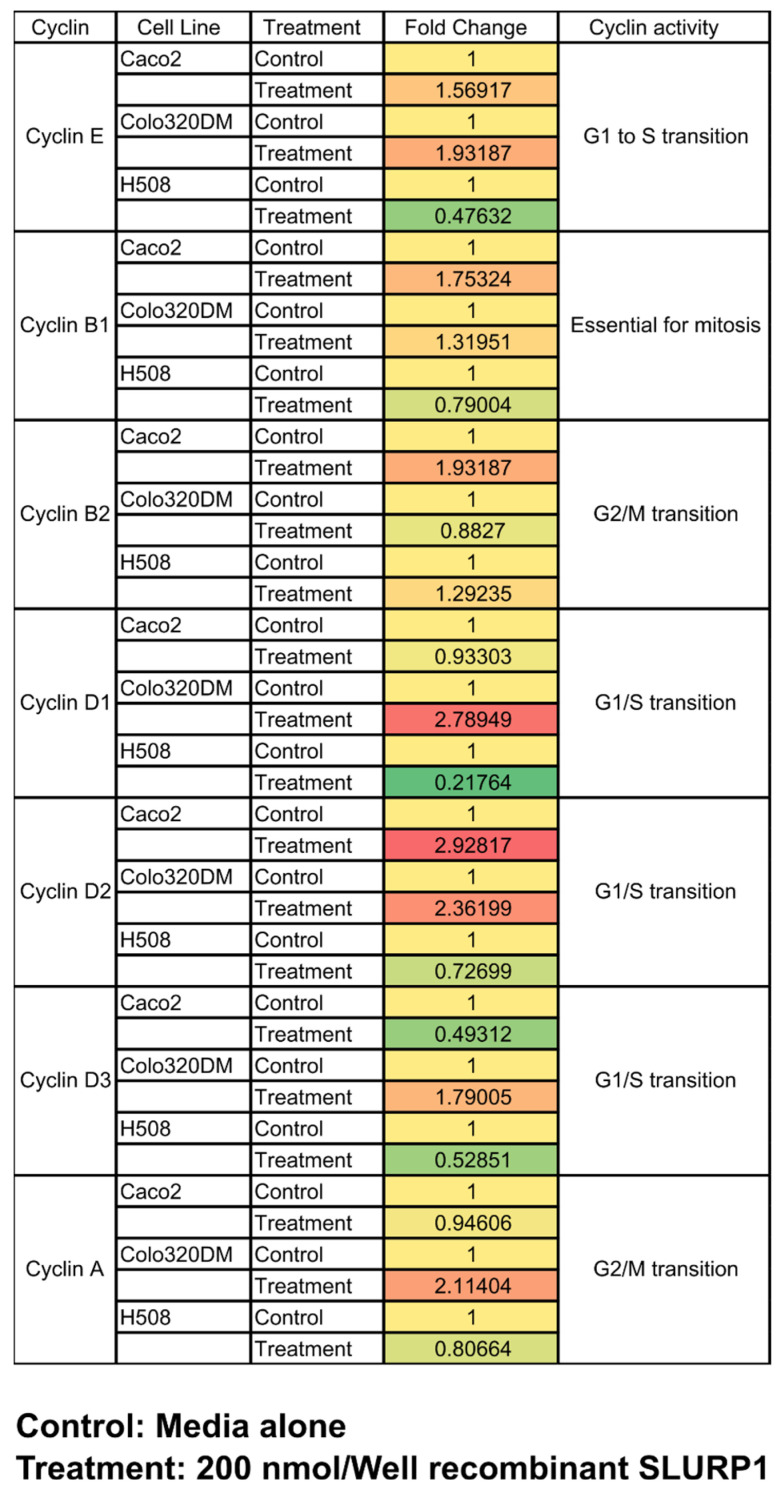
Effect of SLURP1 on cell cycle checkpoints. The expression response of cell cycle checkpoints was assessed by quantitative real-time PCR after SLURP1 treatment in Caco2, Colo320DM, and H508 cell lines. Expression of each candidate gene was presented with the non-treated control. The color intensity indicates the fold change, with red indicating a higher level of expression and green indicating a suppression in comparison to the control (yellow).

Cell cycle arrest by SLURP1 protein should be evident at the mRNA level. qRT-PCR analysis was conducted on Caco2, Colo320DM, and H508 cell lines to confer the effect of SLURP1 in an expression of genes related to the cell cycle. The qRT-PCR analysis of cell cyclin gene expression revealed modulation of cyclins E, B1, and B2 genes, whereas cyclins D1, D2, D3, and A exhibited significant up-regulation, particularly in Colo320DM cells. Gene expression profile reveals SLURP1 prevents cells from entering the M phase; however, it did not affect protein synthesis and cell enlargement (Figure 6). Further, a positive correlation between cell cyclins profile and FACS-based cell cycle assessment, as demonstrated for reduced G2 population in SLURP1-treated Caco2 cells, is evidence for the effective role of SLURP1 in controlling the cell signaling to maintain the cell cycle.

### 3.7. Tumor Regression

**Figure 7:**
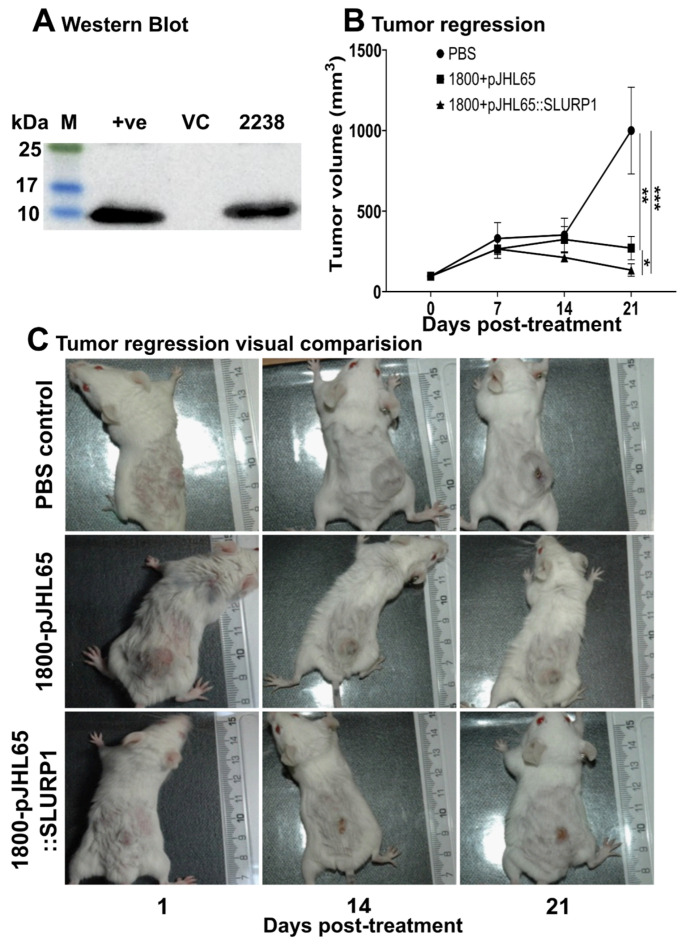
Recombinant protein expression and in vivo anti-tumor implementation of SLUP1 delivered by *Salmonella* Typhimurium (ST). (**A**) Western blot confirming the expression of rSLURP1. The purified rSLURP1 protein expressed by *E. coli* (DE3) was used as a positive (+ve) control. Vector control (VC) represents ST JOL1800 with an empty vector, and JOL2238 denotes therapeutic strain. (**B**) Tumor volume in BALB/c mice post-challenged with a therapeutic vector or ST JOL1800 with empty vector or PBS was monitored for 21 days. Data were analyzed by Student’s *t*-test, and the significant differences were represented, * *p* < 0.05, ** *p* < 0.01, and *** *p* < 0.001, *n* = 8. The error bar indicates mean ± SD. (**C**) Prominent regression of tumor was demonstrated in mice inoculated with ST delivering rSLURP1.

**Figure 8:**
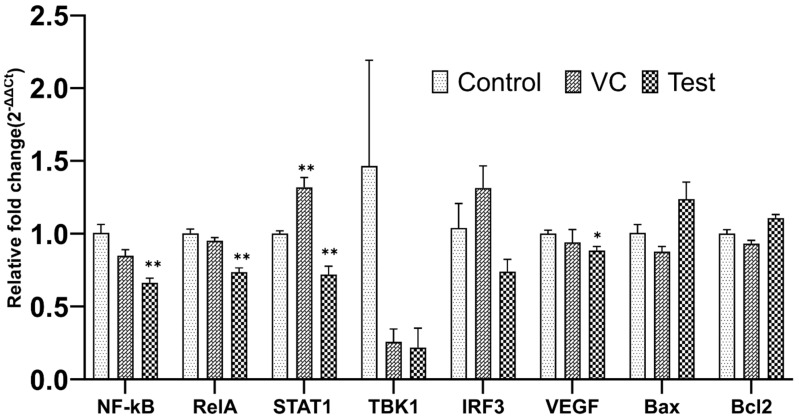
Expression of genes in the tumor. Relative expression of cell cycle-related and tumor-related genes was quantified by qRT-PCR. Data were analyzed by Student’s *t*-test against the non-treated control, and the significant difference was determined at * *p* < 0.05 and ** *p* < 0.01, *n* = 5. The error bar denotes mean ± SD.

There was a significant sequence homology between human SLURP1, mice SLURP1, and several other neurotoxins, such as α-Bungarotoxins (Figure S1). Hence, we hypothesized that the effect of human SLURP1 could still be present in mice models. To evaluate this concept, we cloned human SLURP1 into pJHL65 antigen secretion plasmid and transformed it into an attenuated *Salmonella* strain to deliver it directly into the tumor microenvironment. For the maximum therapeutic outcome of SLURP1, it should act upon the surface of cancer cells where the nAChRs are distributed in abundance. Thus, the invasion of the ST strain into the extracellular spaces of the tumor matrix is essential. The efficient secretion of the therapeutic protein was facilitated by the *bla* signal sequence at the N-terminus of the protein, and its secretion efficacy was confirmed by Western blotting (Figure 7A) [28,29,30]. The uncropped blots and molecular weight markers are shown in Supplementary Figure S2. The CT26 tumor-bearing mice inoculated with the therapeutic strain or the strain with empty plasmid or PBS were monitored for changes in tumor volume for three weeks post-booster inoculation. Significant tumor regression was observed in all ST-treated mice (Figure 7B). Six of the eight mice treated with the rough ST JOL1800 strain secreting SLURP1 demonstrated a substantial reduction in tumor volume, retaining only a scar at the tumor implanted site (Figure 7C). The prominent tumor reduction displays the synergistic anti-tumoral effect imposed by the treatment with SLURP1 secretion. Further, the expression of immune modulatory genes assessed in tumors exhibited an anti-inflammatory environment by downregulated expression of NF-κB, RelA, and STAT1 (Figure 8). Down-regulation of vascular endothelial growth factor (VEGF) in treated tumors further supports an anti-angiogenesis property of SLURP1 that helps with tumor reduction.

## 4. Discussion

The potential of nicotinic acetylcholine receptors to induce an anti-inflammatory environment can be exploited as an avenue for novel anticancer treatments [31]. These nAChRs are widely distributed in normal and cancerous cells and act as receptors for various environmental clues that either promote or diminish cancerous outcomes [32,33]. The behavior of these receptors appears to be complicated and can exert opposite outcomes depending on the interacting molecules which may promote pro or anti-oncogenic pathways [34]. Among various potent ligands that interact with nAChRs, the SLURP1 protein gained much interest due to its ability to arrest epithelial cancer cell proliferation via specific interaction with α7-nAChRs [35,36]. Despite both nicotine and SLURP1 interacting with the same α7-nAChR targets, their effects on cancer proliferation are diametrically opposed. Nicotine has been shown to promote cancer growth, whereas SLURP1 exhibits inhibitory properties. Interestingly, SLURP1 could completely reverse pro-oncogenic features exerted by nicotine [37], revealing its strong protective functions behaving as an auto/paracrine signaling molecule. Chronic inflammation serves as a common underlying factor in numerous cancer types, with colon cancer standing as a prominent concern that affects millions of individuals. This necessitates increasing understanding of the intricate relationship between inflammation and colon cancer. While such links could be exploited for therapeutic approaches. Hence, it is pragmatic to investigate the protective functions of SLURP1 on colon cancer cell lines, to establish a plausible link between a reduction in inflammatory responses and cancer cell proliferation.

Growth inhibition assays conducted using colon cancer cell lines, Caco2, Colo320DM, and H508 cells revealed dose-dependent inhibition of cell proliferation and cell migration. Comparing three cell lines, the H508 cells originating from the human cecum demonstrated the highest responsiveness, while Caco2 was the least. In this study, all three cell lines, which were confirmed to stably express α7-nAChRs through qRT-PCR analysis, exhibited a notable temporal upregulation in the endogenous expression of SLURP1 at the mRNA level. Most cancer cells demonstrate increased expression of nAChRs, which is essential to rapid cell proliferation [38]. Additionally, as time progressed, there was a gradual increase in the concentration of SLURP1 in the culture supernatants. The interaction of SLURP1 with its target receptor promotes Ca^2+^ ion influx in a dose-dependent manner, possibly linked to increased expression of α7-nAChR, which are membrane-bound ion channels [39]. In most cancers, nAChRs are upregulated [40,41,42] and perform vital functions in cancer continuation. However, the anti-inflammatory switch that is activated by SLURP1 appeared to enhance its anti-inflammatory signaling cascade to favor anticancer properties.

Nicotinic acetylcholine receptors either can be homomeric (α7 or α9) or heteromeric (α3, α5, α9, α10, β1, β2, and β4) and the SLURP1 found to interact with both α7 and non-α7 nAChRs [8,43]. Studies have revealed that stimulation of nonneuronal cells with rSLURP1 induces signals that can be mediated by intracellular Ca^2+^ [35] and phosphatidylinositol-3-kinase (PI3K) [44,45]. The α7-nAChRs demonstrate a high permeability to Ca^2+^ and Ca^2+^ ions [9,27,33,46]. The activity of SLURP1 on colon cancer cells was essential to have the function of divalent cations, especially Ca^2+^ ions [47]. The expression of α7-nAChRs is essential for continued turnover of the mucocutaneous epithelium and certain cancer types in humans via regulation of genes related to signal transduction, cell cycle regulation, apoptosis, and cell adhesion proteins [31,32,34,45]. The α7-nAChRs downstream regulation leading to the expression of NF-kB has been described in various types of normal and malignant cells [37,48,49]. So, the regulation in α7-nAChRs expression via SLURP1 interaction is anticipated for the control of cell functions, proliferation, and cell cycle.

Considering the induced inflammatory conditions by *Salmonella* Typhimurium LPS, SLURP1 could significantly abort LPS-induced inflammation in all three types of cells, with a substantial reduction in Colo320DM and H508 cells. Inflammatory cytokines TNF-α and IL-1β were significantly downregulated; however, IFN-γ reduction was not significant. Thus, the application of rSLURP1 suppresses the expression of TNF-α and IL-1β, conferred with published reports [50], and supports the anti-inflammatory role. We hypothesize that SLURP1 potentially induces Toll-like receptor 9 (TLR9) endosomal receptor in suppressing TNF-α mediated inflammatory signaling; it is probably linked to the endogenous expression of SLURP1 [50]. The anti-proliferative effect of SLURP1 is evident in the reduction of cell proliferation at higher concentrations (Figure 2). This finding supports the idea that SLURP1 inhibits cell growth. Additionally, the application of SLURP1 counteracts the cell proliferative influence of LPS (Figure 4C); this reinforces the anti-proliferative role of SLURP1, which nullifies the proliferative effects induced by LPS.

To further assure the SLURP1 effect on cell cycle regulation, propidium iodide-mediated flow cytometric assessment of cell cycle arrest, and qRT-PCR mediated quantification of cell cyclins were conducted. At all the tested concentrations of SLURP1, cell cycle arrest at the P1 transition phase was prominent for Colo320DM and H508 cells. Likewise, treatment of Caco2 cells with SLURP1 demonstrated a potential arrest at the G1 phase at higher concentrations only. It was also evident in the qRT-PCR assessment of cell cyclins, where most cyclins regulating G1/S transition were downregulated in H508 cells. However, the expression kinetics of cyclins on Caco2 and Colo320DM cells did not follow the pattern observed with H508 cells. Therefore, SLURP1 must have an underlying signaling pathway that diminishes cancer cell proliferation other than cell cycle arrest.

It is also reported that SLURP1 is present in almost all tissues and organs [7,10,43,51]. Therefore, it can be hypothesized that SLURP1 confers protective/homeostatic function throughout the body and maintains the inflammatory state and related immune-modulatory functions. Receptor targets of SLURP1, such as α7-nAChRs, Plasminogen Activator, Urokinase Receptor (PLAUR), Plasminogen Activator, Urokinase (PLU), and choline acetyltransferases are significantly expressed in primary colon tumors. Furthermore, the expression of SLURP1 and its modulation in human colon cancer can control the state of the tumor [52]. As a result, SLURP1 could exert a profound effect on the colon environment by affecting its receptor targets in a manner that promotes an anticancer environment. Consideration of SLURP1 as a protective protein has also been reported in clinical cohort studies, where it was observed that cancer patients with high levels of SLURP1 in their plasma had a greater chance of surviving than cancer patients with low levels of SLURP1 [15].

Since human SLURP1 has never been tested for its effect on cancer tumors, in the present study, SLURP1’s effect on mice CT26 colon cancer tumor model was evaluated. The conserved amino acid sequence in SLURP1 between humans and mice suggests a potential cross-species effect on mice tumors. To assess the anti-tumor potential of recombinant human SLURP1 in mice, the *Salmonella* delivery system was used. *Salmonella* can accumulate and secrete proteins directly into cancer tumor tissues, making it an effective approach for this study [53]. The effective secretion of proteins was assessed by Western blot analysis. Interestingly, *Salmonella* treatment in tumor-bearing mice causes a significant reduction in tumors, leaving almost complete regression in several treated mice. Even though a profound effect of treatment could be observed, further elucidation must be undertaken to clarify the specific effect caused by the SLURP1 protein. The anti-inflammatory properties of SLUPR1 have been established [50], and that is reflected in the downregulation of NF-κB (*p* = 0.0020), RelA (*p* = 0.0010), and STAT1 (*p* = 0.0032) in tumors of mice treated with it. As a result of this situation, tumors regressed. The expression of the proapoptotic marker, Bax, was not significantly upregulated in the tumor, which might reflect the non-apoptotic control of cancer growth [54]. The treated mice exhibited a significant down-regulation of VEGF expression (*p* = 0.0178), a phenomenon previously reported to correlate with the inhibition of angiogenesis in both in vitro and in vivo settings [55]. The suppression of VEGF in SLURP1-treated tumors is in parallel with published reports [56] that support the anti-angiogenesis property of SLURP1 to reduce tumor proliferation.

The current findings related to SLURP1 antagonizing α7-nAChR could have significant implications for alleviating colorectal cancer by reducing the inflammatory responses. The evidence supporting the reversal of LPS-mediated inflammation through the potentiation of α7-nAChR could have major implications for enhancing the efficacy of anticancer treatments and addressing diseases such as inflammatory bowel disease, for which there is still no safe remedy to alleviate. Fundamental to the SLURP1’s mechanism, we hypothesize its anticancer properties are derived by α7-nAChR mediated anti-inflammatory cascade that inflicts Ca^2+^ channeling and NF-kB mediated cell cycle arrest at the G1 state. In addition, delivery of SLUPR1 through *Salmonella* demonstrates future therapeutic implications for cancer treatment. The insights provided in this study will undoubtedly be beneficial for the development of safe and effective treatment strategies for colorectal cancer, a disease that annually claims the lives of over one million people worldwide.

## Data Availability

Data will be made publicly available upon publication and request for peer review.

## Supplementary Materials

The following supporting information can be downloaded at https://www.mdpi.com/article/10.3390/pharmaceutics15102462/s1, Figure S1: Amino acid sequence alignment comparison of human SLURP1 and a few selected proteins. An amino acid sequence alignment was conducted for human SLURP1, human SLURP2, Human Lynx1, Human Lypd6, Mouse SLURP1, Neurotoxin 1, Erabutoxin, and α- Bungarotoxin. The degree of sequence conservation was also demonstrated; Figure S2. The original uncropped blot image showing all the bands with all molecular weight. Lanes 1 and 6 represent the molecular marker (size range from 10 to 180 kDa). Lanes 2 and 5 denote the purified rSLURP1 protein expressed by *E. coli* (DE3) as a positive control. Lane 3 indicates Vector control (VC) (ST JOL1800 with an empty vector), and lane 4 represents the therapeutic strain, JOL2238. Table S1: Primer sequences used in qRT-PCR.
