# Supplementary material for: Inflammation-Related Immune-Modulatory SLURP1 Prevents the Proliferation of Human Colon Cancer Cells, and Its Delivery by Salmonella Demonstrates Cross-Species Efficacy against Murine Colon Cancer
Source: Pharmaceutics. 2023 Oct 13;15(10):2462. doi: 10.3390/pharmaceutics15102462 (PMC10609686; doi:10.3390/pharmaceutics15102462)
Supplement: Supplementary file 1 [file pharmaceutics-15-02462-s001.zip › pharmaceutics-2558797-supplementary/Supplementary Figure and Table S1.pdf]

Figure S1. Amino acid sequence alignment comparison of human SLURP1 and a few selected proteins. An amino acid sequence alignment was conducted for human SLURP1, human SLURP2, Human Lynx1, Human Lypd6, Mouse SLURP1, Neurotoxin 1, Erabutoxin, and α- Bungarotoxin. The degree of sequence conservation was also demonstrated.

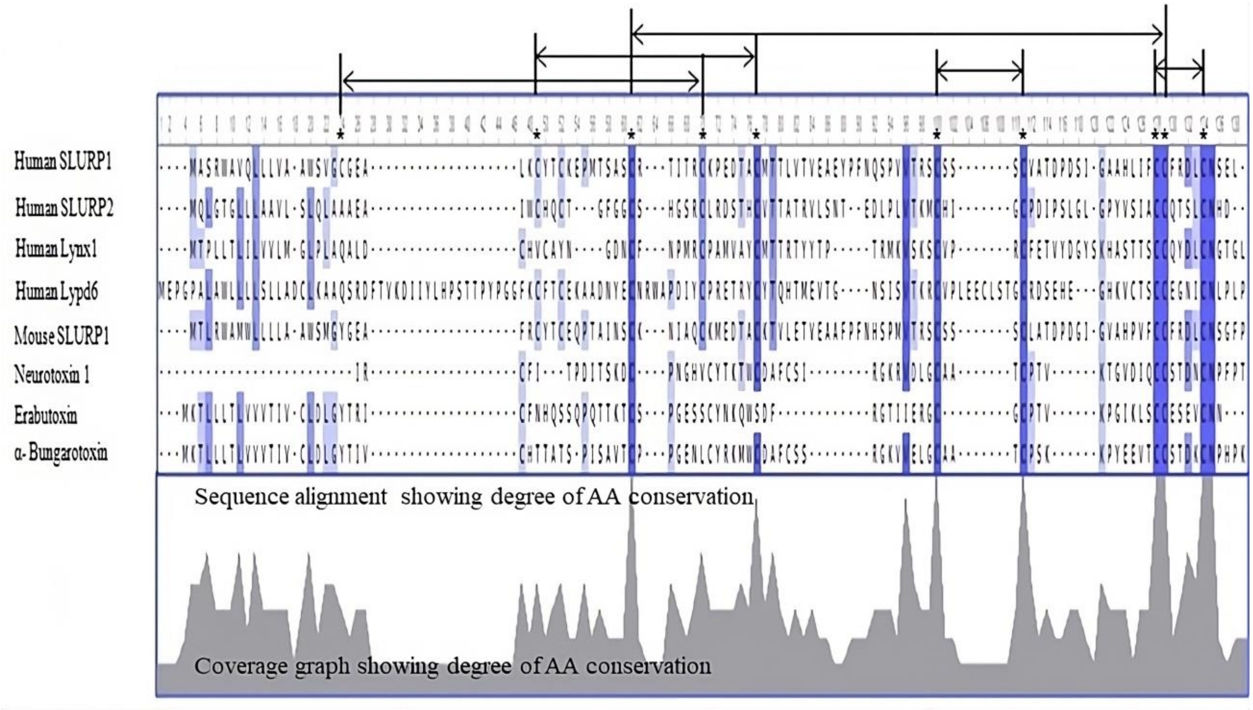

Figure S2. The original uncropped blot image showing all the bands with all molecular weight. Lanes 1 and 6 represent the molecular marker (size range from 10 to 180 kDa). Lanes 2 and 5 denote the purified rSLURP1 protein expressed by E. coli (DE3) as a positive control. Lane 3 indicates Vector control (VC) (ST JOL1800 with an empty vector), and lane 4 represents the therapeutic strain, JOL2238.

1 2 3 4 5 6

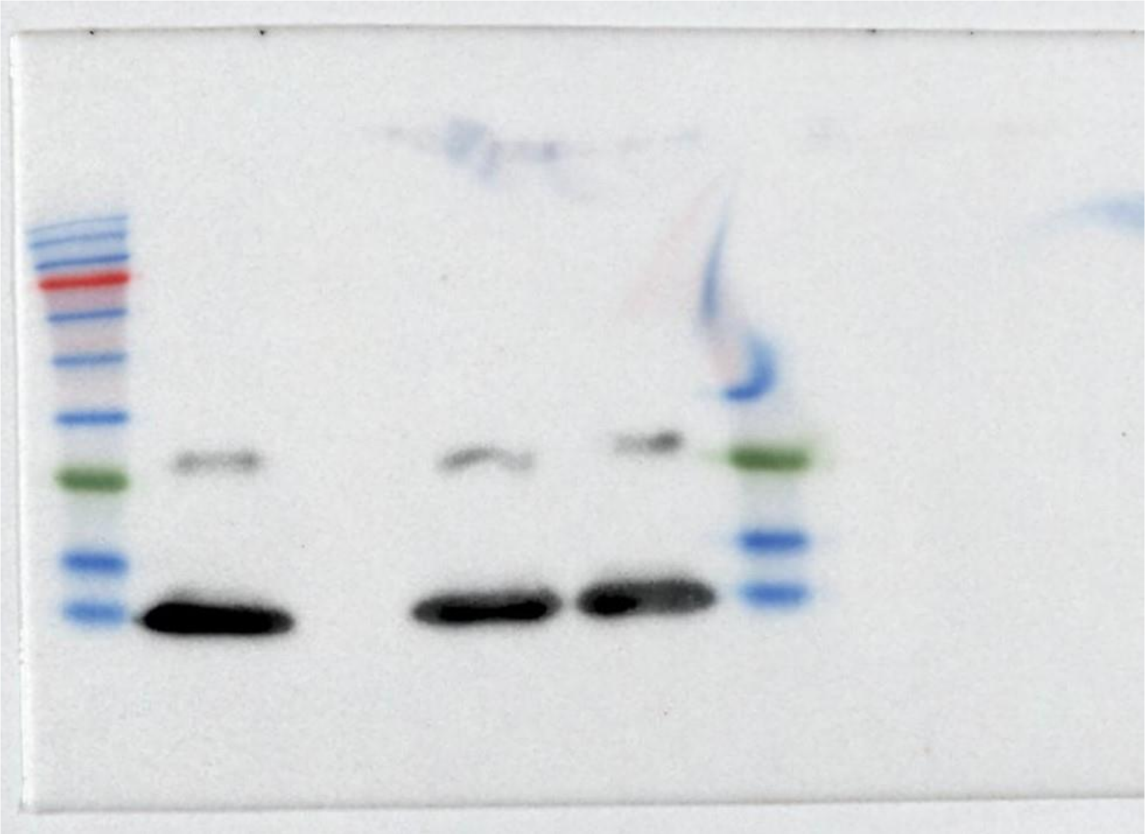

Table S1. Primer sequences used in qRT-PCR.

| Gene | | Forward | Reverse |
|---|---|---|---|
| Cell cycle-related | Cyclin E | GTCCTGGCTGAATGTATACATGC | CCCTATTTTGTTCAGACAACATGGC |
| | Cyclin B1 | GGCTTTCTCTGATGTAATTCTTGC | GTATTTTGGTCTGACTGCTTGC |
| | Cyclin B2 | CCTCCCTTTTCAGTCCGC | CTCCTGTGTCAATATTCTCCAAATC |
| | Cyclin D1 | CATCTACACCGACAACTCCATC | TCTGGCATTTTGGAGAGGAAG |
| | Cyclin D2 | GCTCTGTGCGCTACCGACTT | CACGCTTCCAGTTGCAATCA |
| | Cyclin D3 | TGATTGCGCACGACTTCCT | CAAAGCCTGCCGGTCACT |
| | Cyclin A2 | CTGCATTTGGCTGTGAACTAC | ACAAACTCTGCTACTTCTGGG |
| Endogenous receptor | nAChR | CTGCATTTGGCTGTGAACTAC | CACAAACTGAGACTAAGGCAG |
| (Human) | SLURP1 | TTCTGAGCACGGAGCAATG | TTGCAGGTGTAGCACTTGAG |
| | NF-kB | CTCCACAAGGCAGCAAATAGA | ACTGGTCAGAGACTCGGTAAA |
| | TNF-α | CTTGTTCCTCAGCCTCTTCTC | TCAGCTTGAGGGTTTGCTAC |
| | IFN-γ | GTGGAGACCATCAAGGAAGAC | ACCTCGAAACAGCATCTGAC |
| | IL-1β | CTGCGTGTTGAAAGATGATAAG | CCACATTCAGCACAGGACTC |
| (Mice) | RelA | GGTGGGCACTGGAGTTTATT | GAGGATGGATGGTCCAACTTAC |
| | STAT1 | CTGGAGGAGTTGGAACAGAAA | CTGAATGAGCTGCTGGAAGA |
| | TBK1 | AGTACCCTGCTGCTCTATCA | GTGGACGGTCTCGTTGTAATC |
| | IRF3 | GTCTTAAGGAGCTGTTAGAGATGG | TGGTCAGAGGTAAGGGAGATAG |
| | GAPDH | AACAGCAACTCCCACTCTTC | CCTGTTGCTGTAGCCGTATT |
| | NF-kB | TGGGACCAGCAAAGGTTATT | GATCCCATCCTCACAGTGTTT |
| | VEGF | GCGAAGCTACTGCCGTCC | TCTGCATTGTGATGTTGCTC |
| | Bax | AGGATGCGTCCACCAAGAAGCT | TCCGTGTCCACGTCAGCAATCA |
| | Bcl2 | CCTGTGGATGACTGAGTACCTG | AGCCAGGAGAAATCAAACAGAGG |
