# Supplementary figures and images for: Inflammation-Related Immune-Modulatory SLURP1 Prevents the Proliferation of Human Colon Cancer Cells, and Its Delivery by Salmonella Demonstrates Cross-Species Efficacy against Murine Colon Cancer
Source: Pharmaceutics. 2023 Oct 13;15(10):2462. doi: 10.3390/pharmaceutics15102462 (PMC10609686; doi:10.3390/pharmaceutics15102462)

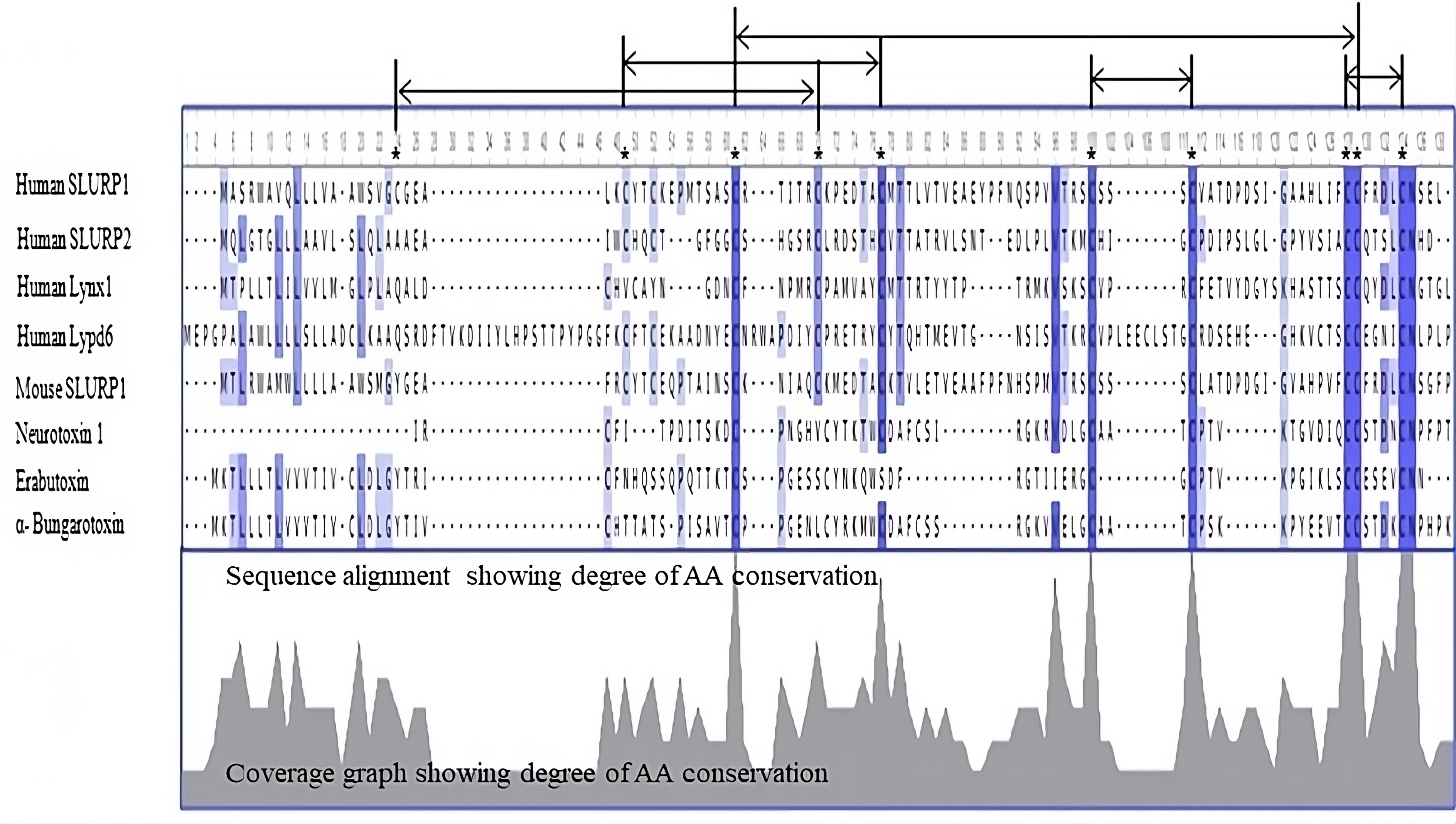

Supplement: Supplementary file 1 [file pharmaceutics-15-02462-s001.zip › pharmaceutics-2558797-supplementary/Figure S1.jpg]

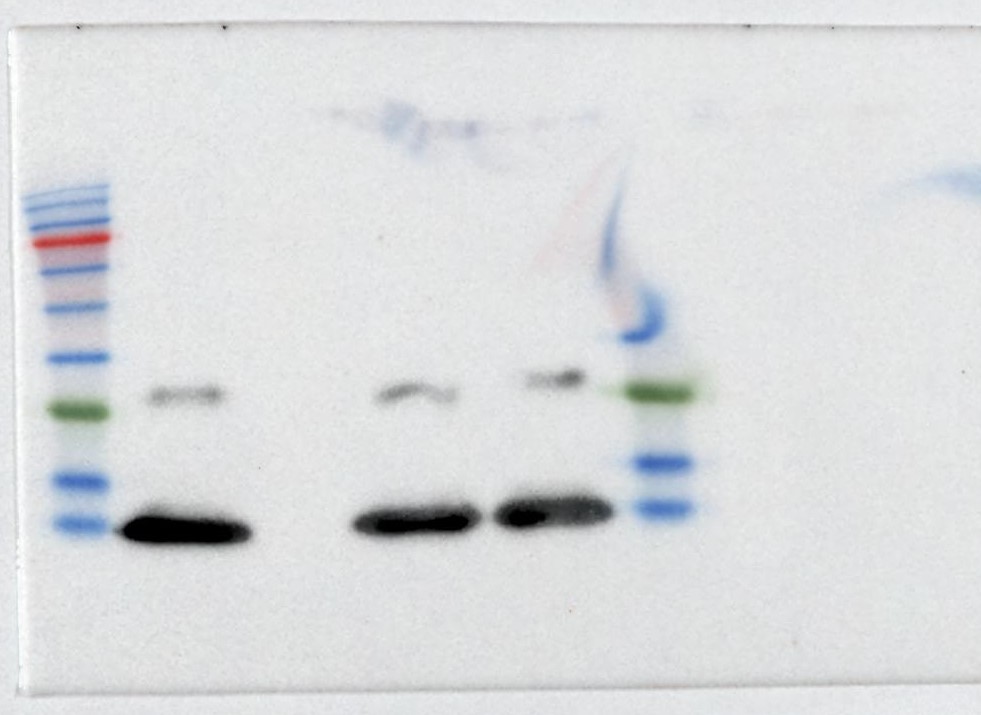

Supplement: Supplementary file 1 [file pharmaceutics-15-02462-s001.zip › pharmaceutics-2558797-supplementary/Figure S2.jpg]
